# Nitrogen has a greater influence than phosphorus on the diazotrophic community in two successive crop seasons in Northeast China

**DOI:** 10.1038/s41598-021-85829-8

**Published:** 2021-03-18

**Authors:** Jing Zhou, Mingchao Ma, Dawei Guan, Xin Jiang, Nianxin Zhang, Fengyue Shu, Yong Kong, Jun Li

**Affiliations:** 1 School of Life Sciences, Qufu Normal University, Jining, 273165 People’s Republic of China; 2 Institute of Agricultural Resources and Regional Planning, Chinese Academy of Agricultural Sciences, Beijing, 100081 People’s Republic of China; 3 Laboratory of Quality & Safety Risk Assessment for Microbial Products (Beijing), Ministry of Agriculture and Rural Affairs, Beijing, 100081 People’s Republic of China

**Keywords:** Microbiology, Ecology, Environmental sciences

## Abstract

Fertilizer-induced changes in soil nutrients regulate nitrogen (N) fixation in the terrestrial biosphere, but the influences of N and phosphorus (P) fertilization on the diazotroph communities in successive crop seasons were unclear. In this study, we assessed the effects of N and P (high vs. low doses) on the abundance and structure of N_2_-fixation communities after wheat and soybean harvest in a long-term (34 and 35 years) fertilization experiment. In both seasons, long-term N addition significantly decreased the abundance of *nif*H genes and 16S rDNA; in addition, high doses of N and P fertilizer decreased the richness of diazotrophs, whereas low doses did not. The proportion of the dominant genus, *Bradyrhizobium*, in the soybean season (86.0%) was higher than that in the wheat season (47.9%). Fertilization decreased diazotroph diversity and the relative abundance of *Bradyrhizobium* in the wheat season, but had insignificant effects in the soybean season. The addition of N, but not P, significantly changed the communities of both diazotrophs (at the genus level) and rhizobia (at the species level) in the two seasons. Soil pH was positively associated with *nif*H abundance and diazotrophic richness; soil NO_3_^−^ content was negatively correlated with diazotrophic richness and positively correlated with diversity. Soil pH and NO_3_^−^ content were the two main drivers shaping the soil diazotrophic community. Overall, long-term inorganic N had a greater influence than P on both diazotrophic abundance and community composition, and diazotrophic diversity was more clearly affected by fertilization in the wheat season than in the soybean season.

## Introduction

Nitrogen (N) is the most essential element limiting productivity in terrestrial ecosystems^1^. The global N input into agricultural systems from synthetic fertilizer has increased more than 40-fold since 1930^2^. According to the National Bureau of Statistics of China, in Heilongjiang Province in Northeast China, where the total grain output was 75.05 billion kg in 2018, accounting for more than 10% of China's total grain output, erosion of its iconic black soil occurrs because of excessive application of chemical fertilizer (http://www.xinhuanet.com). The use of such large amounts of N, organic fertilizer, and organic fertilizer combined with chemical fertilizer has posed severe challenges to the soil microbial community, including bacteria^3^, fungi^4,5^, acidobacteria^6^, arbuscular mycorrhizal fungi^7,8^, diazotrophs^9^, and ammonia oxidizing archaea^10^, in black soil in northeast China.

Biological N fixation is an important ecological process^11^ on earth that is responsible for fixation of as much as 100 Tg N year^−1^ from the atmosphere globally, thus contributing more than 97% to the N input in natural terrestrial ecosystems^12^. Only microorganisms in the bacterial and archaeal domains are known to be capable of fixing atmospheric dinitrogen, a process termed diazotrophy, and *nif*H is a suitable marker for investigating the diversity and composition of diazotroph communities^13^. In agricultural systems, diazotrophs are sensitive to fertilizer management practices^14^, and large amounts of fertilizer might relegate N fixers to second place and may have long-term consequences for diazotrophs and ecosystem processes in the future^15^. Long-term chemical N fertilizer application has been shown to drastically decrease N fixation^11^, decrease diversity, and alter the community structure^16^ and assembly processes^17^ of soil diazotrophs. Taxa in the genera *Bradyrhizobium* and *Burkholderia* have been shown to have a positive association, whereas *Geobacter* and *Anaeromyxobacter* have been shown to have a negative association, with N fertilization for four decades^11^.

Soybean is the most important leguminous food crop^18^ and is often used as a rotation crop because it is excellent in improving soil structure and fertility^19^. Rainfed wheat-soybean-maize rotations account for more than half of China’s food production, particularly in northeast China^20^. The abundance and composition of the diazotrophic community in the soil are related to several factors, including fertilization regimes^11^, soil moisture and temperature^21^, and vegetation types^22^. Soil physicochemical properties are affected by specific plant species through litterfall, root phenes and exudates containing different nutrients, which influence the soil microenvironment and subsequently affect the diazotrophic community^23^. However, the differences in the diazotrophic community abundance and composition between soils in two continuous crop seasons (wheat and soybean, a nonlegume and legume, respectively) under N fertilization regimes are unclear.

Here, we investigated the response of the diazotrophic community assembly in wheat and soybean seasons to five long-term (34 and 35 years) inorganic fertilizer treatments. One approach was no fertilizer added (CK), and the other four approaches involved the addition of artificial fertilizer treatments: low N (N_1_), low N plus low P (N_1_P_1_), high N (N_2_) and high N plus high P (N_2_P_2_). We performed pyrosequencing and real-time PCR based analysis of *nif*H sequences obtained from long-term fertilization trials located at the Heilongjiang Academy of Agricultural Sciences, China. In this study, using experimental gradients of N and P, we addressed the following specific questions: (i) Can N and P addition have different effects on the abundance and composition of the soil diazotrophic community and the dominant phyla/classes/genera in the wheat and soybean seasons? (ii) would shifts in specific bacterial taxa correspond to the fertilization regimes or plants?

## Results

### Effects of long-term fertilization on the abundance of bacteria and diazotrophs

**Figure 1:**
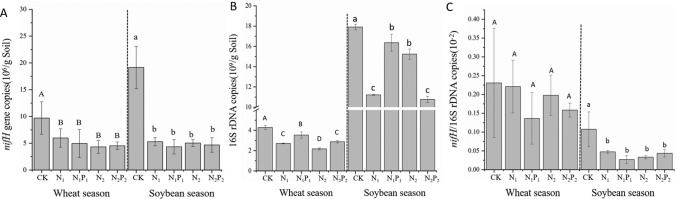
Absolute abundances of the *nif*H gene and 16S rDNA in two crop seasons. (**A**) The absolute abundance of the *nif*H gene. (**B**) The absolute abundance of 16S rDNA; (**C**) The ratio of nitrogen-fixing microorganisms to bacteria.

The abundances of the *nifH* gene (Fig. 1A) and 16S rDNA (Fig. 1B) were all significantly lower in the N input soils (N_1_, N_1_P_1_, N_2_, and N_2_P_2_) than in unfertilized soils in the two seasons. The diazotroph to bacteria ratio showed no clear differences among the five samples in the wheat season but was lower in N input soils (N_1_, N_1_P_1_, N_2_, and N_2_P_2_) than in unfertilized soil in the soybean season (Fig. 1C).

### Effect of fertilization on diazotrophic diversity

**Figure 2:**
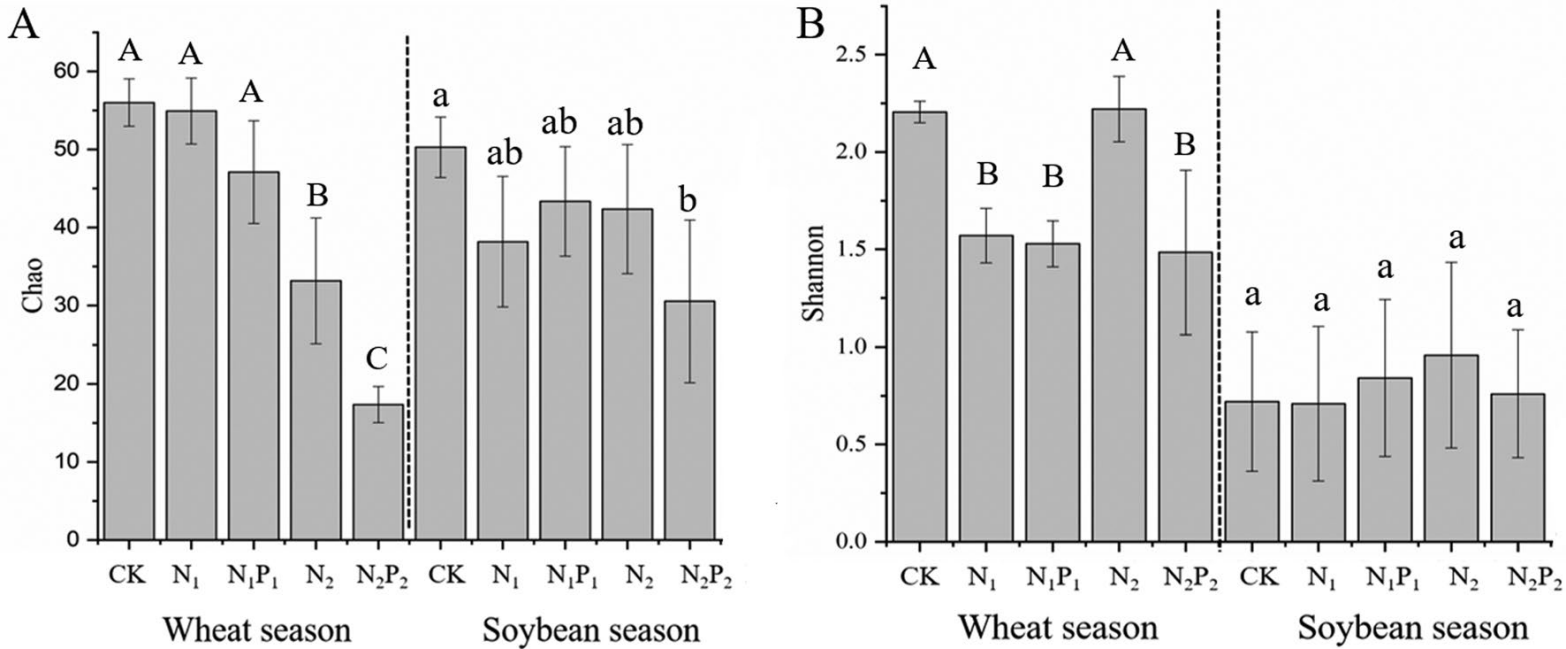
α diversity indices of nitrogen-fixing microorganisms in soil samples under different fertilizer treatments. (**A**) Chao index, (**B**) Shannon index.

A significantly lower Chao index of the *nifH* communities was measured with high fertilizer treatments (N_2_ and N_2_P_2_), whereas the results were not significantly different between low fertilizer treatments (N_1_ and N_1_P_1_) and unfertilized soil in the two seasons (Fig. 2A). Fertilization was considered to have an insignificant effect on the Shannon index at the *P* < 0.05 level (*P* = 0.625) in the soybean season, whereas the index was lower with N_1_, N_1_P_1_, and N_2_P_2_, as compared with CK, in the wheat season (Fig. 2B). The Chao and Shannon indices were lower with N_2_P_2_ than with N_2_ in the two seasons.

### Relationship between soil chemical properties and α-diversity and gene copies

A *t*-test comparison across all treatments in both crop seasons revealed a significant decline in soil pH (Table 1). In contrast, the concentrations of NO_3_^−^, Avail P, Avail K, TN, and OM, which were clearly higher in soil with fertilizer treatments (N_1_, N_1_P_1_, N_2_, and N_2_P_2_) than in unfertilized soil (Table 1). Ammonium (NH_4_^+^) was greater under theN and P fertilizer treatments than CK, whereas no statistically significant differences were observed for plots fertilized during the soybean season, regardless of fertilization (Table 1).

**Table 1:** Soil chemical properties of different fertilizer samples in wheat and soybean seasons.

Season	Treatment	pH	NO_3_^−^ (mg kg^–1^)	NH_4_^+^ (mg kg^–1^)	Avail P (mg kg^–1^)	Avail K (mg kg^–1^)	TN (g kg^–1^)	OM (g kg^–1^)
Wheat	CK	6.36 ± 0.02a	7.67 ± 0.06e	33.83 ± 1.1b	9.27 ± 0.31d	176.63 ± 7.88ab	1.2 ± 0.01c	27.26 ± 0.43b
	N_1_	5.64 ± 0.02b	8.47 ± 0.06d	36.9 ± 2.51ab	10.87 ± 0.64d	183.23 ± 20.47ab	1.28 ± 0b	28.26 ± 0.92ab
	N_1_P_1_	5.59 ± 0.06b	10.5 ± 0.17c	34.77 ± 0.31b	70.83 ± 0.83b	152.17 ± 9.19b	1.38 ± 0.04a	29.86 ± 0.1a
	N_2_	4.64 ± 0.02c	24.53 ± 0.12a	40.73 ± 1.16a	15.27 ± 0.31c	216.57 ± 39.93a	1.42 ± 0.03a	29.47 ± 0.99a
	N_2_P_2_	4.79 ± 0.02c	22.27 ± 0.23b	39.07 ± 1.72a	85 ± 0.72a	143.8 ± 6.71b	1.38 ± 0.03a	29.89 ± 0.31a
Soybean	CK	6.48 ± 0.06A	2.36 ± 1.02B	34.85 ± 0.57A	1.78 ± 0.22D	177.96 ± 7.16B	1.48 ± 0.02ABC	25.75 ± 2.72B
	N_1_	5.47 ± 0.12B	5.09 ± 0.45AB	48.44 ± 11.68A	3.84 ± 0.67D	185.1 ± 4.5B	1.28 ± 0.08C	27.87 ± 0.75B
	N_1_P_1_	4.68 ± 0.19C	11.63 ± 3.42A	42.6 ± 3.01A	27.78 ± 0.22C	200.5 ± 3.71A	1.51 ± 0.09AB	29.96 ± 1.74AB
	N_2_	5.62 ± 0.28B	5.62 ± 1.68AB	41.7 ± 9.32A	64.85 ± 5.51B	174.33 ± 5.9B	1.39 ± 0.01B	27.79 ± 0.96B
	N_2_P_2_	4.9 ± 0.02C	8.85 ± 4.43AB	42.85 ± 12.53A	202.59 ± 13.62A	177.3 ± 5.15B	1.62 ± 0.12A	33.03 ± 1.93A

Values are mean ± standard deviation (*N* = 3). Values within the same column followed by different letters indicate significant difference (*P* < 0.05).

Avail P indicates available phosphorus, Avail K is available potassium, TN is total N and OM is organic matter.

Soil pH showed a highly significant (*P* < 0.01) and positive linear relationship with the number of *nif*H gene copies (r = 0.64, Supplementary Fig. S1A) and the Chao index (r = 0.628, Supplementary Fig. S1C). The oil NO_3_^-^ content had a significant (*P* < 0.01) negative relationship with the number of 16S rDNA copies (r = − 0.595, Supplementary Fig. S1B) and the Chao index (r = − 0.59, Supplementary Fig. S1D), but had a significant (*P* < 0.01) and positive relationship with the Shannon index (r = − 0.527, Supplementary Fig. S1E). Other soil properties including the soil content of NH_4_^+^, Avail P, Avail K, TN and OM were not significantly associated with α diversity indices, and *nif*H gene or 16S rDNA copy numbers.

### Regional distributions in the diazotrophic populations

**Figure 3:**
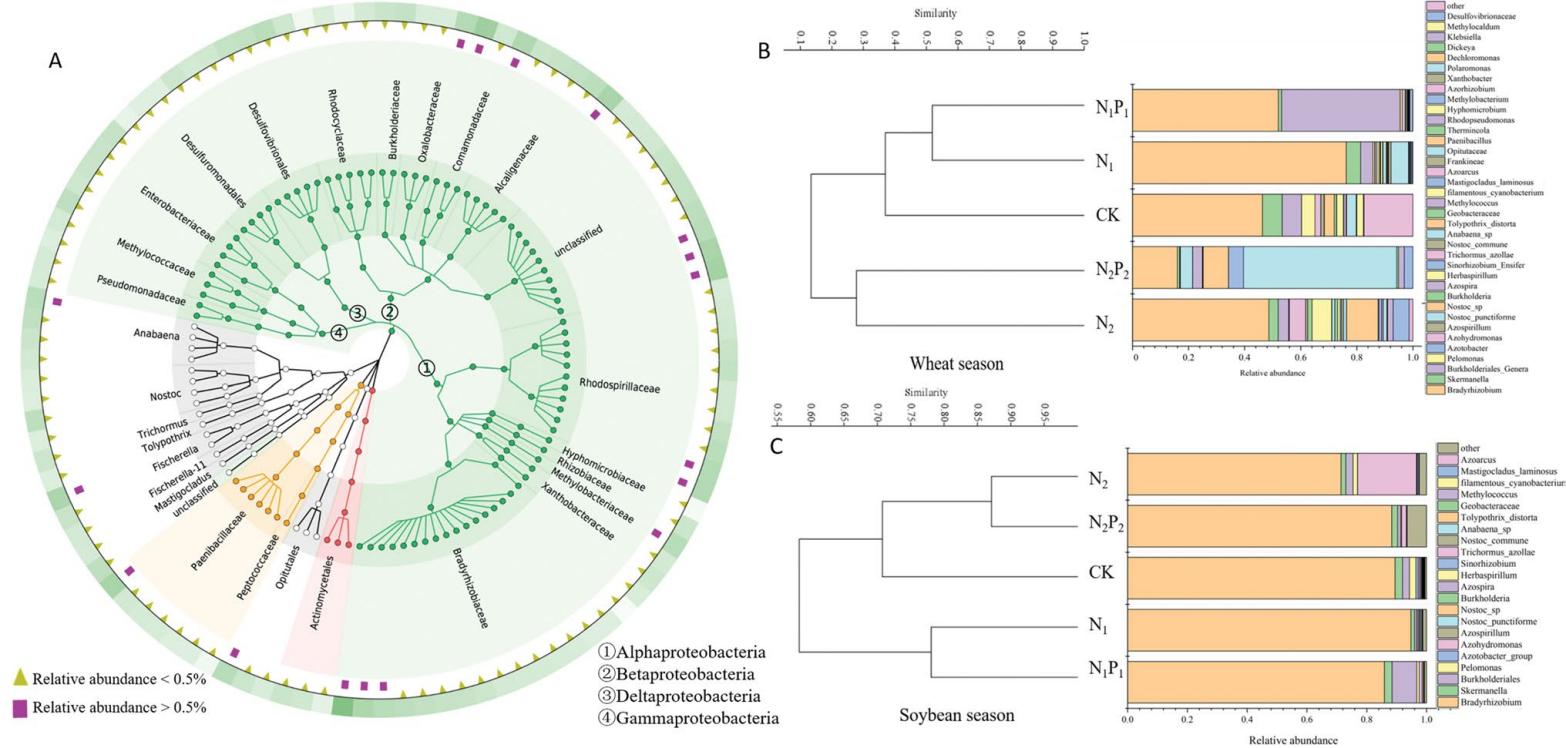
Phylogenetic tree and clustering analysis based on nitrogen-fixing microorganisms. (**A**) Phylogenetic tree displaying the taxonomic information on soil diazotrophic phylotypes. (**B**,**C**) show the results of clustering analysis based on nitrogen-fixing microorganisms (at the genus level) in soils of the wheat and soybean seasons, respectively.

We constructed a phylogenetic tree with the dominant diazotrophic phylotypes, which were generated for the 180,600 *nif*H sequences collected from the 30 soil samples. A large number of the *nif*H gene sequences were affiliated with Proteobacteria, which accounted for 86.5% of the bacteria in wheat soil and 98.5% in soybean soil (on average), followed by Cyanobacteria (0.13–27.8%), Firmicutes (0–11.26%) and Verrucomicrobia (0–6.2%) (Supplementary Table S3). For the phylum Proteobacteria, the classes Alphaproteobacteria, Betaproteobacteria, Deltaproteobacteria, and Gammaproteobacteria, were dominant in all soil samples (Fig. 3 A ①, ②, ③ and ④, respectively). The dominant family was Bradyrhizobiaceae, which was more abundant in the soybean season (71.5–89.5%) than in the wheat season (16.04–76.3%) (Supplementary Table S3).

**Figure 4:**
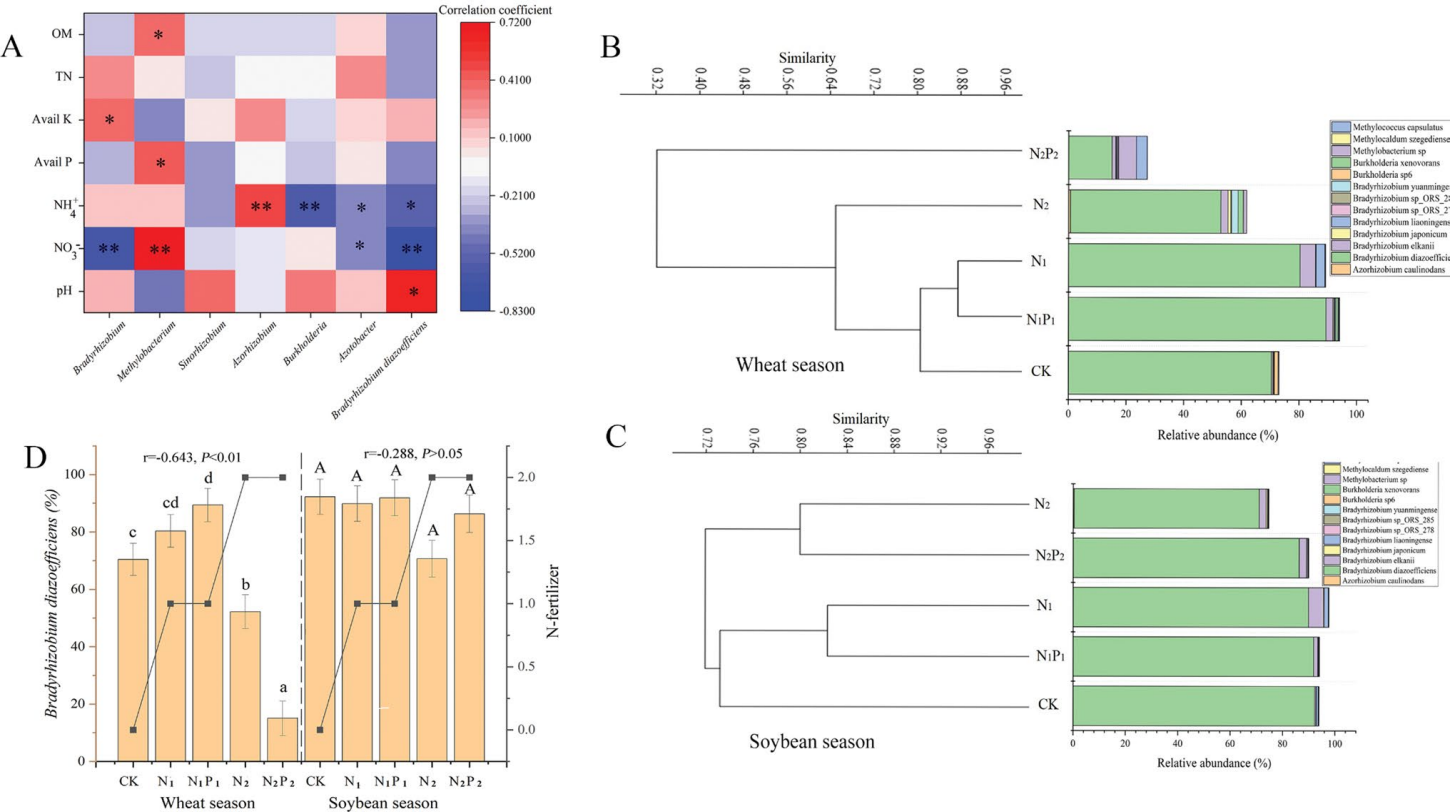
Correlation and cluster analysis at the species level. (**A**) Correlationships between microbial abundance, genera belonging to rhizobia and soil chemical properties. (**B**,**C**) show the results of clustering analysis based on rhizobia (at the species level) in soils of the wheat and soybean seasons, respectively. (**D**) Shows the relative abundance of Bradyrhizobium diazoefficiens in soil samples and their correlation with N fertilizer. *W* soil samples in the wheat season, *S* soil samples in the soybean season.

We found six genera belonging to rhizobia: *Bradyrhizobium*, *Methylobacterium*, *Ensifer* (formerly *Sinorhizobium*), *Azorhizobium*, *Burkholderia,* and Azotobacter (Fig. 4A). Interestingly, the average abundance of the genus *Bradyrhizobium* was much higher in the soybean season (86.0%) than in the wheat season (47.9%) (Supplementary Table S4). In the wheat season, *Bradyrhizobium* abundance under N_1_, N_1_P_1_, and N_2_ was 64.4%, 12.2% and 4.8% higher, respectively, whereas it was 65.4% lower under N_2_P_2_ than CK (Supplementary Table S4). However, there was no clear difference in *Bradyrhizobium* abundance in the soybean season (Supplementary Table S4). The soil NO_3_^-^ content was negatively correlated with the relative abundances of *Bradyrhizobium* (r = − 0.627, *P* < 0.01) and Azotobacter (r = − 0.371, *P* < 0.05)*,* but positively (r = 0.716, *P* < 0.05) correlated with the relative abundance of *Methylobacterium* (r = 0.716, *P* < 0.01) (Fig. 4A)*.* The soil NH_4_^+^ content was negatively correlated with the relative abundance of *Burkholderia *(r = − 0.612, *P* < 0.01*)* and Azotobacter (r = − 0.390, *P* < 0.05)*,* and positively correlated (r = 0.516, *P* < 0.05) with *Azorhizobium* (Fig. 4A)*.*

The abundance of the dominant species, *Bradyrhizobium diazoefficiens*, was negatively correlated with soil NO_3_^−^ (r = − 0.563, *P* < 0.05) and NH_4_^+^ (r = − 0.764, *P* < 0.01) contents, and positively associated with soil pH (r = − 0.636, *P* < 0.05) (Fig. 4A). In addition, the relative abundance of this species was significantly and negatively correlated with the N*-*level in the wheat season, whereas no significant relationship was observed in the soybean season (Fig. 4D)*.*

The clustering results showed that the *nif*H communities were significantly separated by N addition treatments in the wheat (Fig. 3B) and soybean (Fig. 3C) seasons at the genus level. Similarly, the rhizobial group also showed the same trend at the species level (Fig. 4B,C). In the wheat season, compared with CK, *Bradyrhizobium* (Fig. 3B) and *Bradyrhizobium diazoefficiens* (Fig. 4D) were significantly higher in the presence of low N but lower in the presence of high N. However, no significant difference was observed among samples in the soybean season.

### NMDS at the OTU level

The NMDS results revealed that *nif*H community composition varied significantly (*P*< 0.05) with respect to N addition but not P addition. Across the two seasons, the phylogenetic structure of the *nif*H communities shifted in similar ways. The *nif*H communities in soils in the wheat season did not differ significantly from those in the soybean season (Figure S2). Three separate groups were clearly observed along NMDS1 (accounting for 67.31% of the variation in *nif*H community): no fertilized soils (SCK and WCK, orange circles); low N and low N plus P fertilized soils (SN_1_, SN_1_P_1_, WN_1_, and WN_1_P_1_, green circles); and high N and high N plus P fertilized soils (SN_2_, SN_2_P_2_, WN_2_, and WN_2_P_2_, red circles, except for SN_2_ and SN_2_P_2_) (Supplementary Fig. S2). As the amount of added N increased, communities became more different from those in unfertilized soils in both seasons.

### Environmental effects on diazotrophs

**Figure 5:**
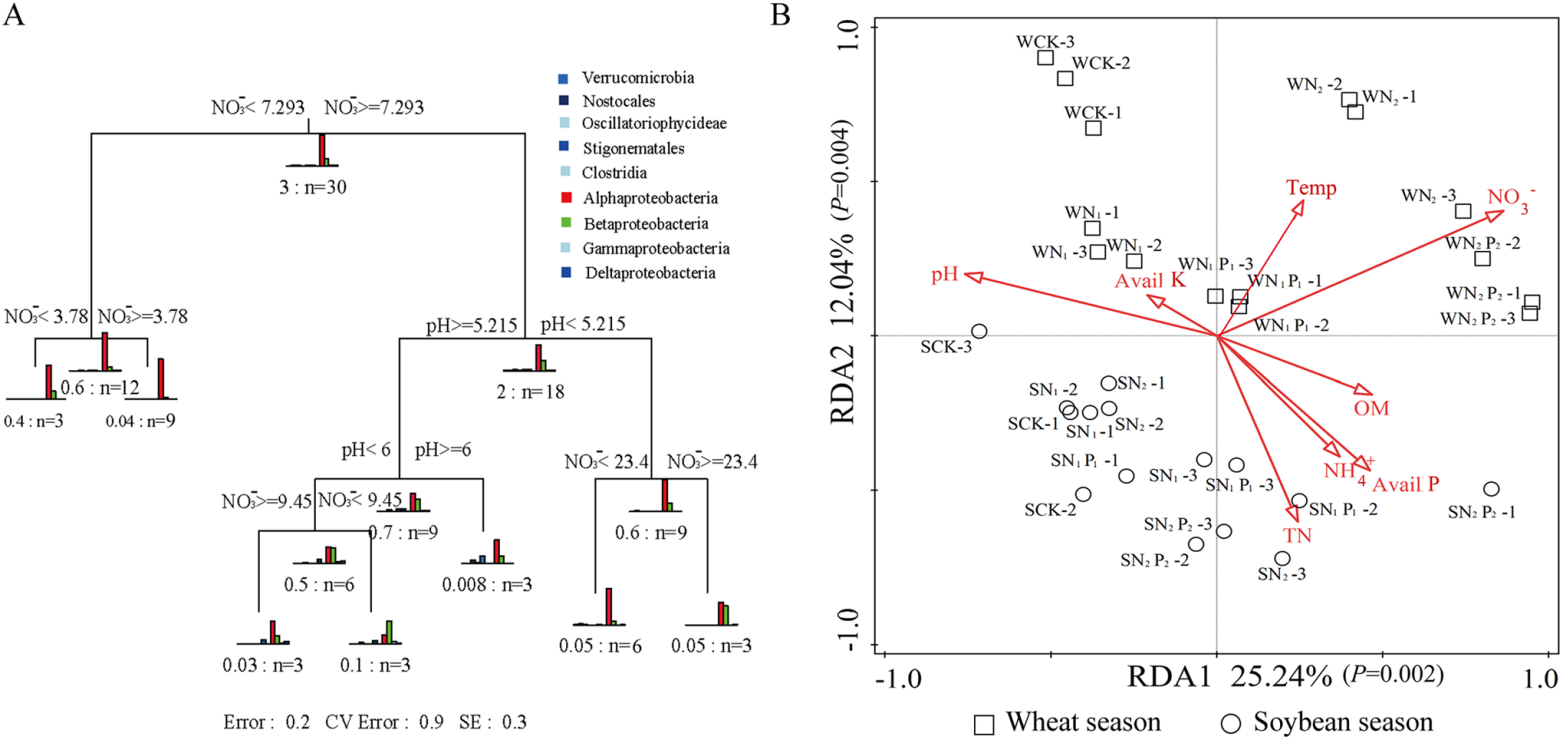
The relationship between the N-fixing community composition and soil chemical properties. (**A**) Multivariate regression tree (MRT) analysis was performed to interpret the relationship between the nitrogen-fixing community composition and soil chemical properties at the class level. (**B**) Redundancy analysis (RDA) was performed to assess the relationship between the nitrogen-fixing community composition and soil chemical properties at the genus level. *W* soil samples in the wheat season, *S* soil samples in the soybean season.

In the MRT, the dominant lineages were first split by NO_3_^−^ content, which explained 26.0% of the variation in community structure (Fig. 5A). The tree explained 71.1% of the variance in the standardized diversity indices. At the second node, the split was determined by soil pH, which explained 17.8% of the variation. The communities were then split by pH and NO_3_^−^, accounting for 14.3% and 8.4% of the variation in the data, respectively (Fig. 5A). The results of RDA with Monte Carlo permutation tests showed that NO_3_^−^, pH, Avail P, and Avail K were significantly (*P *< 0.05) correlated with the changes in the composition of the N-fixing community, with contributions of 41.4%, 17.1%, 13.2%, and 9.2%, respectively (Supplementary Table S5). All samples were separated into two groups (wheat and soybean season: squares and circles, respectively) (Fig. 5B) along the RDA2 axis, except for SCK-3. Along the RDA1 axis, *nif*H communities under low N, and low N plus low P were clearly different from those under CK, and those under high N, and high N plus high P were more different in the two crop seasons. We found that climate factors (temperature and precipitation) were not significantly (*P *> 0.05 in MRT, *P *= 0.05 in RDA) correlated with the changes in the composition of the N-fixing community

## Discussion

### Fertilization decreased bacterial and diazotrophic abundance

The correlation between *nif*H abundance and fertilizer inputs was controversial in previous studies. In our study, the abundance of *nif*H in fertilizer treatments decreased in the two seasons, in agreement with conclusions reached by Zhang^24^ and Coelho^25^ validating Fan's^11^ hypothesis that N fixation and fixers would become less abundant over time in fertilized environments. Urea addition in the present study may have been particularly detrimental for obligate N fixers; their ability to downregulate fixation was limited, and thus they exhibited relatively narrow growth tolerance. For the first time, we reported that the diazotroph to bacteria ratio was more sensitive during the growth and development of soybean than that of wheat. The application of N fertilizers is generally expected to decrease the dependence of the ecosystem on free-living N- fixers^26^, and this finding might explain the slight decrease in *nifH*/16S rDNA in the wheat season. Although symbioses between some N fixing bacteria with soybean can provide N-fixers with an exclusive niche and contribute to their growth^27^, we found a clear decrease in *nif*H/16S rDNA in the soybean season, thus indicating that the symbiotic ability of N-fixers with the soybean decreased under a 35 year N fertilization regime. We concluded that the clear differences in this ratio in the two seasons could be explained by the interactions among individual plant strategies, in agreement with Sheffer's^28^ conclusion. *Nif*H abundance under N_1_ and N_2_ did not show clear differences from those under N_1_P_1_ and N_2_P_2_, respectively, thus indicating that P did not have a significant effect on *nif*H gene copy number.

Most studies have found that an increase in N decreases soil pH^29,30^, and we found the same result; however, P had no such effect. In this study, we found that with increased acidification of black soil, the growth and reproduction of N-fixers was significantly inhibited. Other studies have found that the copy number of the *nif*H gene is strongly positively associated with soil NH_4_^+^^31^ and available K^32^, but negatively correlated with total N^31^.

### Effects of fertilization on diazotrophic diversity

Many studies have found that N addition increases the richness of diazotrophs^33,34^. However, some studies have found that N has no such clear influence^35^. However, we observed significantly lower Chao index values for N fixers under the addition of high N, and high N plus high P in the two seasons (Fig. 2A). One possible reason for this finding is that N fixers have a strong advantage in N deficient conditions, but some species have difficulty surviving and may even die under intense increases in the concentration of available N in the soil microenvironment^35^.

Coelho’s^36^ study on another nonlegume crop, sorghum, showed that the diazotrophic Shannon diversity under high levels of N was lower than that under low levels of N, in agreement with the results in the wheat season in our study. However, the higher proportion *of* Bradyrhizobiaceae in the soybean season than in the wheat season might explain the lower diversity of *nif*H sequences in the soybean season. Furthermore, nodules formed by *Bradyrhizobium* and soybean roots increase the tolerance to various stresses, such as salt^37^, acidity^38^, drought^39^, insecticide^40^, and high aluminium^38^, thus potentially also explaining why *nif*H diversity was not significantly affected by N in the soybean season but was significant in the wheat season. We additionally found that P had clear inhibitory effects on the richness and diversity of diazotrophs.

Shannon indices of diazotrophic and nitrite-dependent anaerobic methane oxidation bacteria^41^ were positively correlated with NO_3_^−^ content, thus indicating that the increase in NO_3_^−^ was beneficial to the diversity of N cycling microorganisms. Santoscaton^42^ found that NO_3_^−^ loads are associated with bacterial 16S rDNA abundance but not *nif*H gene abundance, similar to our results.

### N fertilizer affects the structural composition of N-fixing bacteria

N but not P fertilizer had significant effects on diazotrophic community composition, which indicated that the level of N fertilizer was the most important factor affecting the structural composition of N-fixing bacteria in the black soil of Northeast China. This result was highly consistent with the response of diazotrophic bacterial^25^, ammonia-oxidizing archaeal^43^, bacterial^44^ and fungal^5^ communities to N fertilization regimes.

The process of nitrification in soil is performed partly by gram-negative bacteria in the family Bradyrhizobiaceae, in a process involving the conversion of NH_4_^+^ into NO_2_^-^ and subsequently NO_3_^–^^45^. Therefore, the higher average concentration of NH_4_^+^ in soybean (42.08 mg kg^−1^) than in wheat (37.06 mg kg^−1^) soils, may lead to an increase in Bradyrhizobiaceae in the soybean season. The high abundance of Bradyrhizobiaceae in the soybean season could also be explained by stable symbiosis between leguminous plants (soybean) and rhizobia, although the roots of nonleguminous plants (wheat) can be colonized by rhizobia^25^. Furthermore, linear relationships between the cultivar and the bacterial community have been reported, such as genotype associations of maize with *Azospirillum*^46^, alfalfa cultivars with *Sinorhizobium*^47^, and sorghum cultivars with *Paenibacillus*^48^. Therefore, the results presented here emphasize the importance of cultivar type in selecting N-fixing strains for use as wheat and soybean inoculants.

### N fertilizer affects the structural composition of rhizobia

Rhizobia, a collective name for the symbiotic N-fixing bacteria associated with legumes, comprise 14 genera^1^, six of which were found in the current study. The community structure of rhizobia was distinguished by N levels. *Bradyrhizobium* was reported to be more adapted to acidic soils^49^, while we found a lower abundance under N_2_P_2_ with a lower pH (4.79). Thus, we propose that *Bradyrhizobium* may use suitable amounts of available N to support their growth, whereas N fixation and N fixers will become increasingly less important when NO_3_^-^ is excessive. This hypothesis is based on the negative correlation between *Bradyrhizobium* and the soil NO_3_^−^ content (Fig. 4A). Ahmed^50^ concluded that soil NO_3_^−^ has a negative effect on the activity of N-fixing rhizobia by inhibiting the function of the enzymes nitrogenase and leghaemoglobin.

The dominance of *B. diazoefficiens* over *Bradyrhizobium* sp., *B. japonicum,* and *B. elkanii* revealed a unique community structure of soybean rhizobia in the black soil, a finding not consistent with those of Yan^51^. The negative correlation between *Bradyrhizobium diazoefficiens* and N fertilizer in the wheat season rather than in the soybean season may be explained by the sensitivity of certain bacterial species present in plant types to N fertilizer. In addition, the relatively higher content of NO_3_^−^ under N_2_ and N_2_P_2_ in the wheat season may have caused *Bradyrhizobium diazoefficiens* to become increasingly less important. The abundance of *Bradyrhizobium diazoefficiens* in the wheat season indicated that it is a genospecies whose growth is clearly inhibited by N fertilizer.

### Effects of soil properties on the diazotrophic community

Researchers have confirmed that soil physicochemical characteristics affect the activity of N-fixers^52^. MRT and RDA results confirmed that the soil NO_3_^−^ content was the most important contributor to the soil diazotroph community, a finding consistent with reports by Yang^53^ and Zou^54^. Moreover, soil NO_3_^−^ content was identified as an important predictor of 16S rDNA gene abundance and the α-diversity of the diazotroph community (Supplementary Fig. S1). Neutral or slightly acidic soil conditions are conducive to biological N fixation^18^, while the strong acidity in high N and P may be a severe problem for N fixation, because in such environments legume nodules fail to form, and some rhizobia become inactive^18^. Seminal work by Wang^16^ highlighted the importance of soil pH as a fundamental driver of the distribution of the diazotrophic community, and we reached the same conclusion. In this study, soil acidification in black soil in northeast China, caused by high levels of N fertilization, usually leads to problematic nutrient deficiency or mineral toxicity during N fixation^55^. These findings may aid in predicting the response and feedback of the diazotroph community in farmland ecosystems to high levels of N fertilization.

More recently, researchers have shown that diazotroph diversity and richness are mainly influenced by soil available P^53^ and available K^16^; our results again validated these conclusions. Our findings indicated that soil nutrient availability, which was highly responsive to fertilizer input, was crucial for the establishment of the soil diazotrophic community structure^56^ in black soil in Northeast China.

## Conclusion

Our work provided solid evidence, after 34 and 35 years of experiments, that N fertilization largely influenced diazotroph communities in the soil in two successive crop seasons in northeast China. N is likely to have a greater influence than P on diazotrophic bacteria. The community structure of N-fixing bacteria and rhizobia was clearly associated with the level of N fertilizer. The lower diazotrophic abundance under N fertilizer treatments may have diminished the capacity for biological N fixation in the two seasons. N had greater effects on diazotrophic diversity and the relative abundance of the dominant genus *Bradyrhizobium* in the wheat season than in the soybean season. The different response patterns of diazotrophic abundance, community composition, and diversity to the soil properties revealed a complicated mechanism underlying the diazotrophic population's adaptation to long-term N and P fertilization in two crop seasons. However, we only conducted research at the DNA level, and future research will determine the impact of N fertilization on the functional diversity of diazotrophs in two seasons based on mRNA profiling of *nif*H genes.

## Materials and methods

### Experimental design and sample collection

**Figure 6:**
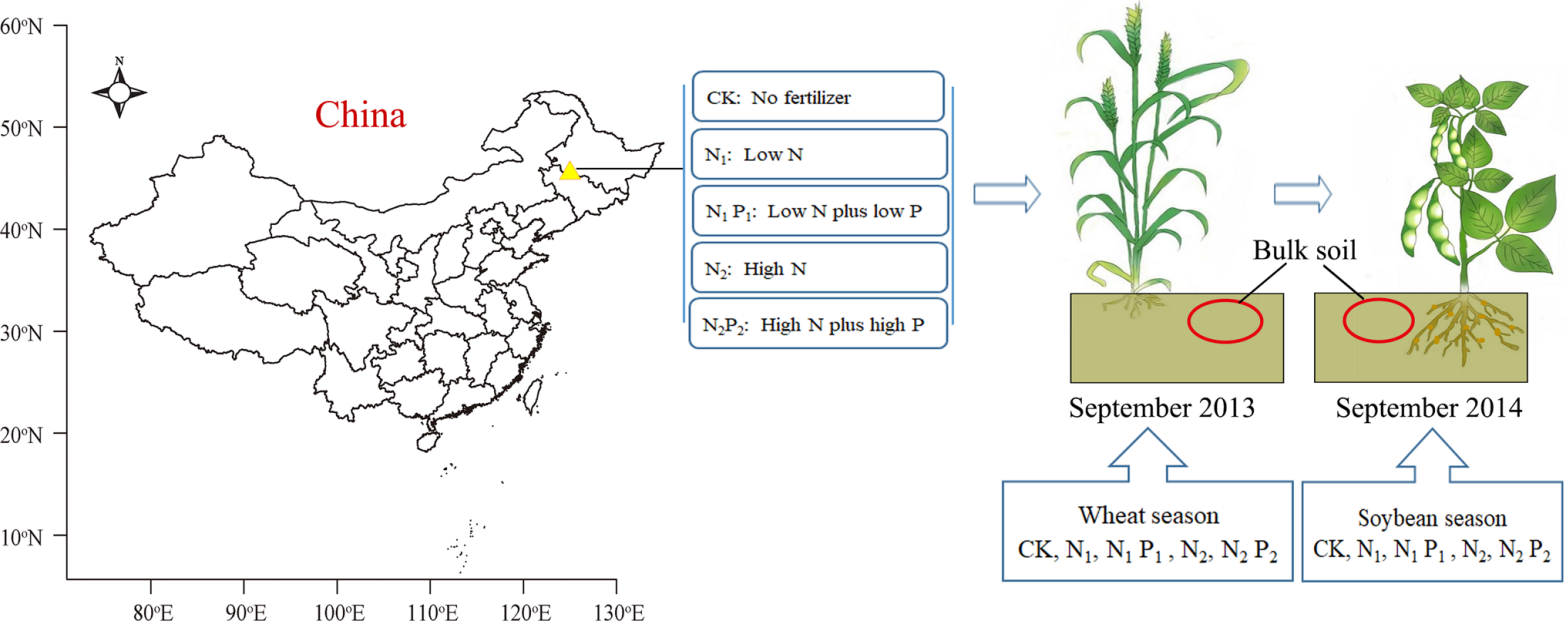
Experimental design and sample collection.

The experimental site was set up in 1979 in Harbin city, Heilongjiang Province, China (45° 40ʹ N, 126° 35ʹ E and altitude 151 m), which is in the secondary terrace of the Songhua River. The soil type is black soil, and the parent material is flooded loess-like clay. The tillage method is a combination of shallow tillage and deep rotation. The annual crop rotation of wheat, soybean and maize was repeated every 3 years in the field with five fertilization treatments in a completely randomized block design with three replicates: CK (without fertilizer), N_1_ (low N), N_2_ (high N), N_1_P_1_ (low N plus low P) and N_2_P_2_ (high N plus high P). Taking into account the difference in N needs for wheat and soybean, we chose different amounts of low N (75 and 150 kg urea ha^−1^ year^−1^ for wheat and soybean, respectively) and high N (150 and 300 kg urea ha^−1^ year^−1^ for wheat and soybean, respectively) treatments. The detailed types and amounts of fertilizer were shown in Supplementary Table S1. N, P, and K fertilizers are all applied after harvest of the previous crop in autumn (September). We collected soil samples in September 2013 and 2014 after wheat and soybean harvests, respectively before the next fertilization. The annual average soil temperature, 10 cm below the surface of the soil was 7.4 and 5.9 °C, and the annual precipitation was 2262 and 296 mm in 2013 and 2014, respectively^44^. The experimental design and sample collection are shown in Fig. 6 [The map was produced using ‘R (i386 3.1.2, https://www.R-project.org, The R Core Team, 2019)’, with the open source packages ‘maps’, ‘mapdata’, and ‘maptools’^57^]. Soil samples were randomly collected from the plow layer of soil (5–20 cm) and stored as described in our previous research^44^.

### Soil chemical properties

Soil pH was measured at a 1:5 ratio of soil to distilled water (weight/volume). Nitrate (NO_3_^−^–N) and ammonium (NH_4_^+^–N) were extracted from 5 g of air- dried soil with 2 M KCl, steam distillation and titration. The available P (Avail P) was extracted with 0.5 M NaHCO_3_ and determined with the molybdenum blue method. Available potassium (Avail K) was extracted with 1 M ammonium acetate and determined by flame photometry. The organic matter (OM) and total N (TN) were determined according to Strickland and Sollins (1987).

### High-throughput sequencing and bioinformatics analysis

Fast DNA SPIN Kit (MP Biomedicals, Santa Ana, CA, USA) was used to extract DNA from 0.5 g of fresh soil. The *nifH* gene was amplified using the primer pairs *nifH* f and *nifH* r^58^. Primer sets and PCR reactions were as detailed in Supplementary Table S2, and amplification reactions were sequenced on the Illumina MiSeq PE300 platform. The raw reads have been deposited in the National Center for Biotechnology Information Database (SRX 1034826). The *nif*H nucleotide sequences were analysed with the QIIME-1.9.1 pipeline. Briefly, the low quality sequences were discarded, and the remaining sequences were converted to amino acid sequences using the FunGene Pipeline of the Ribosomal Database Project according to Ref.^11^. The sequences encoding proteins that contained termination codons or that did not match the *nif*H protein sequence were removed. Operable classification units (OTUs) were classified with a similarity of 95%. The phylogenetic tree for diazotrophic phylotypes at the OTU level in the ecological clusters was built and visualized with GraPhlAn^11^ based on a logarithmic scale. Hierarchical clustering analysis was performed at the genus and species levels with PAST software (version 3.01, folk.uio.no/ohammer/past/)^59^.

### Quantitative PCR analysis

The abundance of bacterial 16S rDNA and the *nif*H gene were analysed with an ABI 7500 Real-Time PCR detection system with primers 515F-806R^3^ and *nif*H f-*nif*H r. Primer sets, the qPCR amplification system and the thermal programme are detailed in Supplementary Table S2. Plasmid DNA containing 16S rDNA and *nif*H fragments were used for quantitative PCR standards. The specificity was determined by melting curve analysis and agarose gel electrophoresis^60^. The ratio of N-fixing microorganisms to bacteria was calculated according to the *nif*H gene and 16S rDNA copy numbers.

### Statistical analysis

Analysis of variance was performed with a randomized complete block design in IBM SPSS Statistics 21. Linear regression analysis was performed to test for statistical significance and the strength of associations between soil chemical properties and α-diversity and gene copy numbers (16S rDNA and *nif*H) in Origin 2020. Phylogenetic tree was visualized using GraPhlAn2^61^ with the data of OTU representive sequences and OTU abundant table. On the basis of Bray–Curtis similarity distance, nonmetric multidimensional scaling (NMDS) was used to analyse the *nif*H community structure at the OTU level. A multivariate regression tree (MRT) analysis was performed with the package “mvpart” in the “R” statistical program to interpret the main relationships between the biological data (at the class level) and environmental factors [soil chemical properties and climate status (temperature and precipitation)]^62^. The correlations between the N-fixing communities (at the genus level) and environmental factors were determined with redundancy analysis (RDA), by using CANOCO 5.0. A logarithmic transformation was performed to normalize the data and the significance (*P*-value) for the first two canonical axes was evaluated by means of Monte Carlo tests based on 999 permutations.

## Supplementary Information

Supplementary Information.
