# Supplementary material for: Nitrogen has a greater influence than phosphorus on the diazotrophic community in two successive crop seasons in Northeast China
Source: Sci Rep. 2021 Mar 18;11:6303. doi: 10.1038/s41598-021-85829-8 (PMC7973567; doi:10.1038/s41598-021-85829-8)
Supplement: Supplementary file 1 — Supplementary Information. [file 41598_2021_85829_MOESM1_ESM.docx]

**Nitrogen has a greater influence than phosphorus on diazotrophic community in two successive crop seasons in northeast China**

Zhou Jing^a^, Ma Mingchao^bc*^, Guan Dawei^bc^, Jiangxin^bc^, Zhang nianxin^a^, Shu Fengyue^a^, Kong Yong^a^, Li Jun^bc*^

^a^ School of Life Sciences, Qufu Normal University, Jining, 273165, China

^b^Institute of Agricultural Resources and Regional Planning, Chinese Academy of Agricultural Sciences, Beijing 100081, China

^c^ Laboratory of Quality＆Safety Risk Assessment for Microbial Products (Beijing), Ministry of Agriculture and Rural Affairs*,* Beijing 100081, PR China

*Corresponding Author: Ma Mingchao and Li Jun: [mamingchao@caas.cn](mailto:mamingchao@caas.cn), [lijun01@caas.cn](mailto:lijun01@caas.cn), Institute of Agricultural Resources and Regional Planning, Chinese Academy of Agricultural Sciences, Beijing 100081, PR China. Tel: +8610 82106208. FAX: +86 1082108702


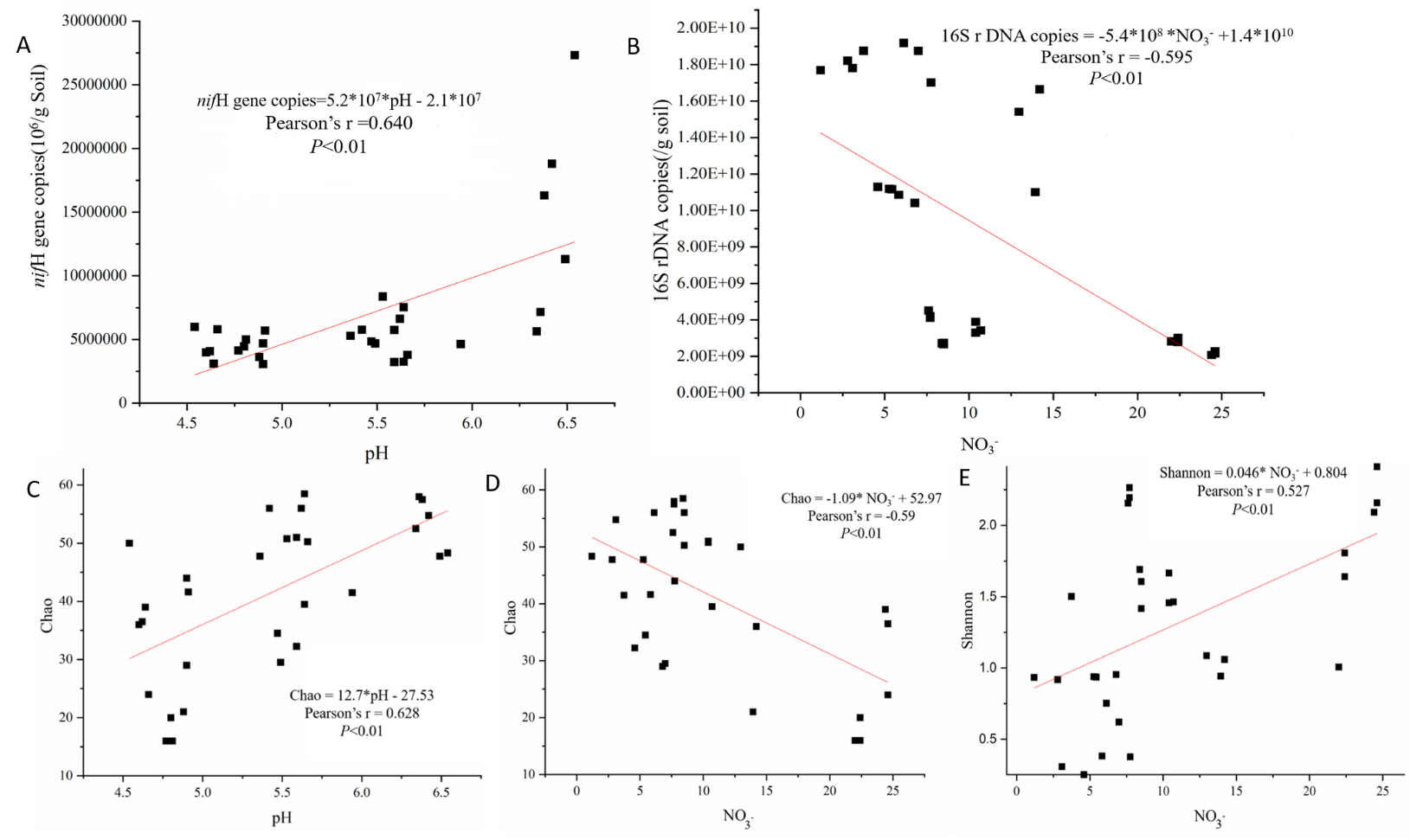


Figure S1 Linear regression relationship of soil chemical properties with gene copies and α diversity index.


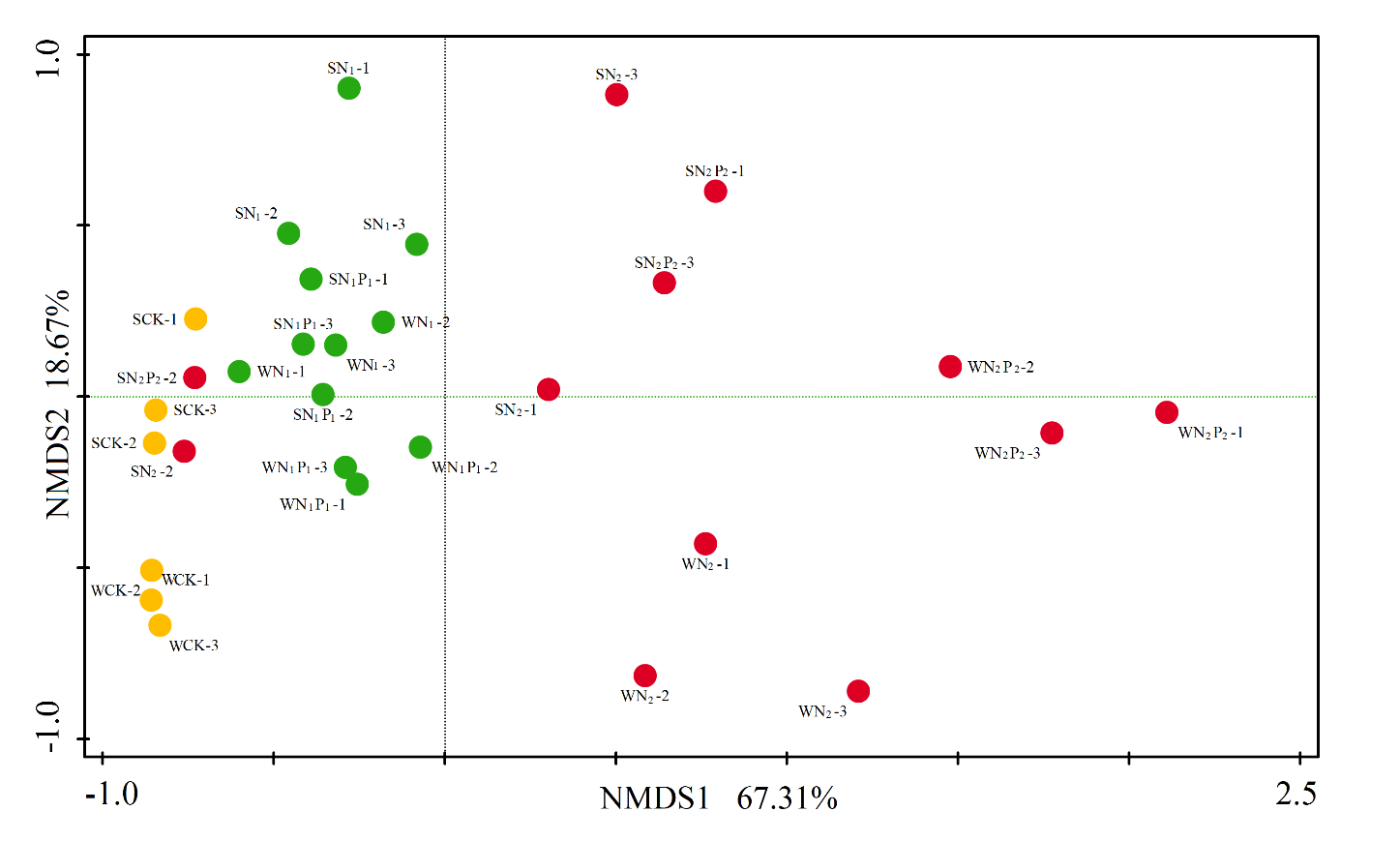


S: soybean season, W: wheat season.

Figure S2 Non-metric multidimensional scaling based on Bray-Crutis distance at OTU level.

Table S1 Five fertilized treatments of the long-term experiment in this study.

| Treatments | Fertilizers^1^ added, (kg/hm^2^· y) | |
| --- | --- | --- |
|  | Wheat season | Soybean season |
| CK | 0 | 0 |
| N_1_ | N 150 | N 75 |
| N_2_ | N 300 | N 150 |
| N_1_P_1_ | N 150, P_2_O_5_ 75 | N 75, P_2_O_5_ 75 |
| N_2_P_2_ | N 300, P_2_O_5_ 150 | N 150, P_2_O_5_ 150 |

^1^N fertilizer was applied as urea while P fertilizer was as calcium super phosphate and ammonium hydrogen phosphate

Table S2 Primer sets and amplification conditions used in this study.

| Primer | Sequence (5’-3’) | Thermal profiles (PCR) | Thermal profiles (qPCR) | 20 μL qPCR reaction mixture |
| --- | --- | --- | --- | --- |
| *nifH* f | AAA GGY GGW ATC GGY AAR TCC ACC AC | 5 min at 95°C , 25 cycles consisting of 60 s at 95°C, 30s at 60°C and 30 s at 72°C, 45 s at 72°C. | 5 min at 95°C , 40 cycles consisting of 30 s at 95°C, 30 s at 60°C and 32 s at 72°C. | 10 μL SYBR®Premix Ex Taq (TliRNaseH Plus, 2×, Takara Bio, Japan), 0.2 μL PCR forwardand reverse primer (both 20 μM), 1μL DNA template (containing 10–20 ng total DNA) and 8.6 μL double distilled water (ddH_2_O). |
| *nifH* r | TTG TTS GCS GCR TAC ATS GCC ATC AT |  |  |  |
| 515F | GTGCCAGCMGCCGCGGTAA | 94 °C for 2 min,  30 cycles of 94°C for 30 s, 50 °C for 30 s and 72 °C for 1 min of extension, followed by 72 °C for 6 min | 5 min at 95°C , 40 cycles consisting of 30 s at 95°C, 30 s at 60°C and 32 s at 72°C. | 10 μL SYBR®Premix Ex Taq (TliRNaseH Plus, 2×, Takara Bio, Japan), 0.2 μL PCR forwardand reverse primer (both 20 μM), 1μL DNA template (containing 10–20 ng total DNA) and 8.6 μL double distilled water (ddH_2_O). |
| 806R | GGACTACVSGGGTATCTAAT |  |  |  |

Table S3. The average abundances (%) of the 5 phyla and 16 families under long-term fertilizer treatments.

| Level |  | Wheat season | | | | | Soybean season | | | | |
| --- | --- | --- | --- | --- | --- | --- | --- | --- | --- | --- | --- |
|  | name | CK | N_1_ | N_1_P_1_ | N_2_ | N_2_P_2_ | CK | N_1_ | N_1_P_1_ | N_2_ | N_2_P_2_ |
| Phylum | Actinobacteria | 0±0 | 0.91±0.05 | 0.15±0.06 | 0.78±0.61 | 0.06±0.1 | 0±0 | 0.3±0.27 | 0±0 | 0.02±0.03 | 0.03±0.03 |
|  | Verrucomicrobia | 0.01±0.01 | 6.21±2.17 | 0.2±0.2 | 1.06±0.65 | 0±0 | 0±0 | 0.46±0.52 | 0.04±0.08 | 0±0 | 0±0 |
|  | Cyanobacteria | 27.8±4.76 | 1.58±0.76 | 0.13±0.11 | 3.69±3.41 | 4.61±7.42 | 1.29±1.08 | 0.48±0.59 | 0.09±0.08 | 0.27±0.24 | 0.23±0.4 |
|  | Firmicutes | 0.04±0.05 | 0±0 | 0.02±0.02 | 11.26±1.74 | 9.04±4.76 | 0±0 | 0.02±0.02 | 0.01±0.02 | 1.29±1.17 | 2.91±2.77 |
|  | Proteobacteria | 72.16±4.81 | 91.3±1.87 | 99.5±0.29 | 83.21±5.23 | 86.29±8.55 | 98.71±1.08 | 98.74±1.07 | 99.85±0.07 | 98.42±1.11 | 96.83±2.43 |
| Family | Paenibacillaceae | 0.04±0.05 | 0±0 | 0.02±0.02 | 11.26±1.74 | 9.04±4.76 | 0±0 | 0.02±0.02 | 0.01±0.02 | 1.26±1.15 | 2.9±2.77 |
|  | Peptococcaceae | 0±0 | 0±0 | 0±0 | 0±0 | 0±0 | 0±0 | 0±0 | 0±0 | 0.03±0.03 | 0.01±0.02 |
|  | Bradyrhizobiaceae | 46.4±1.46 | 76.27±2.92 | 52.05±10.27 | 48.89±9.3 | 16.04±8.59 | 89.49±6.32 | 94.79±3.4 | 85.92±8.52 | 71.45±33.7 | 88.37±5.51 |
|  | Hyphomicrobiaceae | 0.01±0.01 | 0±0 | 0±0 | 0±0 | 0±0 | 0±0 | 0±0 | 0±0 | 0.01±0.01 | 0.02±0.03 |
|  | Methylobacteriaceae | 0±0 | 0.04±0.03 | 0.18±0.09 | 0.99±0.81 | 5.3±8.34 | 0±0 | 0±0 | 0.04±0.03 | 0.07±0.06 | 0±0 |
|  | Rhizobiaceae | 0.28±0.04 | 0.04±0.03 | 0.03±0.03 | 0.13±0.15 | 0±0 | 0.09±0.12 | 0.02±0.03 | 0.02±0.04 | 0.45±0.63 | 0.01±0.01 |
|  | Xanthobacteraceae | 0±0 | 0.04±0.07 | 0.01±0.01 | 0.53±0.92 | 0±0 | 0±0 | 0.04±0.04 | 0.44±0.58 | 0.36±0.31 | 0±0 |
|  | Rhodospirillaceae | 8.18±2.82 | 6.56±4.34 | 1.44±0.49 | 4.1±4.33 | 0.85±1.25 | 2.9±1.84 | 1.58±0.85 | 3.11±3.28 | 1.89±1.07 | 2±1.8 |
|  | Alcaligenaceae | 2.07±0.39 | 0.5±0.13 | 1.09±0.72 | 5.81±1.21 | 0.18±0.31 | 0.48±0.4 | 0.45±0.27 | 0.9±0.52 | 19.67±33.41 | 1.57±2.11 |
|  | Burkholderiaceae | 0.87±0.4 | 0±0 | 0.43±0.29 | 1.55±1.34 | 0±0 | 0.19±0.15 | 0±0 | 0±0 | 0.08±0.13 | 0.03±0.06 |
|  | Comamonadaceae | 4.86±1.26 | 0.9±0.22 | 0.68±0.16 | 1.53±1.45 | 54.65±19.22 | 2.18±1.51 | 0.12±0.13 | 0.85±0.58 | 1.51±2.54 | 3.5±3.62 |
|  | Oxalobacteraceae | 2.3±2.39 | 0.71±0.13 | 0.03±0.03 | 6.96±11.13 | 0±0 | 0.12±0.2 | 0.02±0.03 | 0.03±0.05 | 0±0 | 0±0 |
|  | Rhodocyclaceae | 0.1±0.02 | 0.78±0.27 | 0.01±0.02 | 0±0 | 0±0 | 0.13±0.11 | 0.55±0.9 | 0.18±0.19 | 0.01±0.01 | 0.01±0.02 |
|  | Enterobacteriaceae | 0.06±0.05 | 0.31±0.27 | 0.02±0.03 | 2.24±0.9 | 2.69±1.49 | 0±0 | 0±0 | 0.02±0.03 | 0.21±0.36 | 0±0 |
|  | Methylococcaceae | 0.2±0.13 | 0.07±0.11 | 0.16±0.07 | 0±0 | 3.51±5.53 | 0.03±0.03 | 0.04±0.08 | 0±0 | 0.11±0.07 | 0.09±0.14 |
|  | Pseudomonadaceae | 0±0 | 0±0 | 0±0 | 0±0 | 0±0 | 0.76±1.31 | 0±0 | 0±0 | 0.05±0.09 | 0.02±0.03 |

Table S4. Average abundances (%) of rhizobia under long-term fertilization regimes.

|  | Wheat season | | | | | Soybean season | | | | |
| --- | --- | --- | --- | --- | --- | --- | --- | --- | --- | --- |
|  | CK | N_1_ | N_1_P_1_ | N_2_ | N_2_P_2_ | CK | N_1_ | N_1_P_1_ | N_2_ | N_2_P_2_ |
| *Bradyrhizobium* | 46.37±1.44 | 76.27±2.93 | 52.05±10.27 | 48.64±8.99 | 16.04±8.59 | 89.49±6.32 | 94.79±3.4 | 85.92±8.52 | 71.38±33.74 | 88.37±5.51 |
| *Methylobacterium* | 0±0 | 0.04±0.03 | 0.18±0.09 | 0.99±0.81 | 5.3±8.34 | 0±0 | 0±0 | 0.04±0.03 | 0.07±0.06 | 0±0 |
| *Sinorhizobium* | 0.28±0.04 | 0.04±0.03 | 0.03±0.03 | 0.13±0.15 | 0±0 | 0.09±0.12 | 0.02±0.03 | 0.02±0.04 | 0.45±0.63 | 0.01±0.01 |
| *Azorhizobium* | 0±0 | 0±0 | 0±0 | 0.53±0.92 | 0±0 | 0±0 | 0.03±0.03 | 0.02±0.03 | 0.32±0.28 | 0±0 |
| *Burkholderia* | 0.87±0.4 | 0±0 | 0.43±0.29 | 1.55±1.34 | 0±0 | 0.19±0.15 | 0±0 | 0±0 | 0.08±0.13 | 0.03±0.06 |
| Azotobacter | 0±0 | 0±0 | 0±0 | 0±0 | 0±0 | 0.76±1.31 | 0±0 | 0±0 | 0.05±0.09 | 0.02±0.03 |

Table S5 Forward selection results of RDA analysis

| Name | Explains % | Contribution % | pseudo-F | *P*-value |
| --- | --- | --- | --- | --- |
| NO_3_^-^ | 21.1 | 37.9 | 7.5 | 0.002 |
| Avail K | 8.2 | 14.7 | 3.5 | 0.002 |
| pH | 4.4 | 8 | 2 | 0.03 |
| Avail P | 4.2 | 7.5 | 1.9 | 0.04 |
| Temperature | 3.0 | 5.3 | 1.5 | 0.05 |
| NH_4_^+^ | 2.9 | 5.2 | 1.4 | 0.172 |
| OM | 2.1 | 3.8 | 1 | 0.438 |
| TN | 2.2 | 3.9 | 1 | 0.414 |
| Precipitation | 1.0 | 2.1 | 0.8 | 0.650 |
